# Vaccines Through Centuries: Major Cornerstones of Global Health

**DOI:** 10.3389/fpubh.2015.00269

**Published:** 2015-11-26

**Authors:** Inaya Hajj Hussein, Nour Chams, Sana Chams, Skye El Sayegh, Reina Badran, Mohamad Raad, Alice Gerges-Geagea, Angelo Leone, Abdo Jurjus

**Affiliations:** ^1^Department of Biomedical Sciences, Oakland University William Beaumont School of Medicine, Rochester, MI, USA; ^2^Department of Anatomy, Cell Biology and Physiology, Faculty of Medicine, American University of Beirut, Beirut, Lebanon; ^3^Lebanese Health Society, Beirut, Lebanon; ^4^Department of Experimental and Clinical Neurosciences, University of Palermo, Palermo, Italy

**Keywords:** vaccines, immunization, history of vaccines, global health

## Abstract

Multiple cornerstones have shaped the history of vaccines, which may contain live-attenuated viruses, inactivated organisms/viruses, inactivated toxins, or merely segments of the pathogen that could elicit an immune response. The story began with Hippocrates 400 B.C. with his description of mumps and diphtheria. No further discoveries were recorded until 1100 A.D. when the smallpox vaccine was described. During the eighteenth century, vaccines for cholera and yellow fever were reported and Edward Jenner, the father of vaccination and immunology, published his work on smallpox. The nineteenth century was a major landmark, with the “Germ Theory of disease” of Louis Pasteur, the discovery of the germ tubercle bacillus for tuberculosis by Robert Koch, and the isolation of pneumococcus organism by George Miller Sternberg. Another landmark was the discovery of diphtheria toxin by Emile Roux and its serological treatment by Emil Von Behring and Paul Ehrlih. In addition, Pasteur was able to generate the first live-attenuated viral vaccine against rabies. Typhoid vaccines were then developed, followed by the plague vaccine of Yersin. At the beginning of World War I, the tetanus toxoid was introduced, followed in 1915 by the pertussis vaccine. In 1974, The Expanded Program of Immunization was established within the WHO for bacille Calmette–Guerin, Polio, DTP, measles, yellow fever, and hepatitis B. The year 1996 witnessed the launching of the International AIDS Vaccine Initiative. In 1988, the WHO passed a resolution to eradicate polio by the year 2000 and in 2006; the first vaccine to prevent cervical cancer was developed. In 2010, “The Decade of vaccines” was launched, and on April 1st 2012, the United Nations launched the “shot@Life” campaign. In brief, the armamentarium of vaccines continues to grow with more emphasis on safety, availability, and accessibility. This mini review highlights the major historical events and pioneers in the course of development of vaccines, which have eradicated so many life-threatening diseases, despite the vaccination attitudes and waves appearing through history.

## Introduction

Vaccines constitute one of the greatest success stories within the health sector. They form part of a multifaceted public health response to the emergence of pandemics. This review is general in nature. It highlights the major historical cornerstones in the development and progress of various types of vaccines since the beginning and through the ages until today.

It recognizes the major pioneers whose work has made a difference in the advancement of this vital health field, despite all the anti-vaccination movements that appeared through the ages. Multiple reviews were encountered during our literature search; however, each of those reviews dealt with a specific aspect of vaccination like effectiveness of a particular vaccine, or side effects of another or even attitudes toward vaccines. Consequently, this work tried to put together the major achievements through history stressing the importance, continuous vital role, and the need for immunization for health prevention and protection as well as its impact on human experience.

The physiological mechanisms behind vaccination are well established. Vaccination activates the immune system and induces both innate and adaptive immune responses thus leading to the production of antibodies, in the case of a humoral response, or to the generation of memory cells that will recognize the same antigen, if there is a later exposure. Periodic repeat injections can improve the efficacy and effectiveness of inoculations (1).

The approval of a vaccine abides by a set of well-established international rules and regulations. Prior to their approval by the respective health authorities, scientists test vaccines extensively in order to ensure their efficacy, safety, and effectiveness. Next to antibiotics, vaccines are the best defense that we have to date against infectious diseases; however, no vaccine is actually 100% safe or effective for everyone. This is attributed to the fact that each body reacts to vaccines differently (2–4). Significant progress has been made over the years to monitor side effects and conduct research relevant to vaccine safety. In addition, vaccine licensing is a lengthy process that may take 10 years or longer. The Food and Drug Administration (FDA) and the National Institute of Health (NIH) require that vaccines undergo the required phases of clinical trials on human subjects prior to any use in the general public. This process is becoming more complex as more caution and care is being allocated to the quality of the market product.

Furthermore, vaccines can be divided into different categories depending on the way that they are prepared including live-attenuated vaccines, inactivated vaccines, subunit vaccines, conjugate vaccines, and toxoids.

## Live-Attenuated Vaccines

Live-attenuated vaccines are used more frequently for viruses rather than bacteria, since the former contain a lesser amount of genes and can be controlled more easily (5). The most common method in formulating live-attenuated vaccines involves passing the virus through successions of cell cultures to weaken it. This will produce a form of the virus that is no longer able to replicate in human cells. However, it will still be recognized by the human immune system, hence protecting the body from future invasions. Examples of such vaccines are measles, rubella, mumps, varicella (more commonly known as chickenpox), and influenza. The disadvantage of using this technique is that the virus may transform into a more virulent form due to a certain mutation and cause illness once injected into the body. Although this rarely occurs, it must always be taken into consideration (6).

## Inactivated Vaccines

By using heat, radiation, or certain chemicals, one can inactivate a microbe. The microbe will no longer cause illness but can still be recognized by the immune system. Poliovirus and Hepatitis A are common examples of inactivated vaccines. This type of vaccine has the disadvantage of being effective for a shorter period of time than live-attenuated vaccines. Multiple boosters of the vaccine are sometimes required to improve effectiveness and sustainability (6).

## Subunit Vaccines

A subunit vaccine contains only portions of the microbe that can be presented as antigens to the human immune system instead of the microbe as a whole. The antigens or the microbe portions that best activate the immune response are usually selected. An influenza vaccine in the form of shots is an example. In addition, a recombinant subunit vaccine has been made for the hepatitis B virus. Hepatitis B genes are injected into maker cells in culture. Once these cells reproduce, the desired antigens of the virus are produced as well, and these can be purified for use in vaccines (6).

## Conjugate Vaccines

Conjugate vaccines are designed from parts of the bacterial coat. However, these parts may not produce an effective immune response when presented alone. Hence, they are combined with a carrier protein. These carrier proteins are chemically linked to the bacterial coat derivatives. Together, they generate a more potent response and can protect the body against future infections. Vaccines against pneumococcal bacteria used in children are an example of conjugate vaccines (6).

## Toxoids

Some bacteria release harmful toxins that cause illness in infected individuals. Vaccinations against such types of bacteria are prepared by inactivating or weakening the toxin using heat or certain chemicals. This will help prepare the immune system against future invasion. The vaccine against tetanus caused by the neurotoxin of *Clostridium tetani* is a good example of a toxoid (6).

## Vaccination: Its Determinants and Modulation with Age

The generation of vaccine-mediated protection is a complex challenge. Effective early protection is conferred primarily by the induction of antigen-specific antibodies. The quality of such antibody responses has been identified as a determining factor of efficacy. Efficacy requires long-term protection, namely, the persistence of vaccine antibodies and/or the generation of immune memory cells capable of rapid and effective reactivation upon subsequent microbial exposure (7).

The exponential development of new vaccines raises many questions about their impact on the immune system. Such questions related to immunological safety of vaccines as well as triggering conditions such as allergy, autoimmunity, or even premature death (7). Such issues were always looked for and monitored and some vaccines were even stopped because of these issues.

Recent vaccine models rely on both a cell-mediated response and a humoral immune response with highly specific antibodies and have shown an adequate amount of success. This, however, has not been the case for a few diseases such as tuberculosis where the humoral immunity mounted by the bacille Calmette–Guerin (BCG), the only currently used human vaccine, is inefficient in conferring proper immunization (8). However, T cells do take part indirectly in the production of antibodies and of secreted biological molecules (e.g., Interferon) for protection. It seems that a proper mounted immunity is better achieved by vaccine-induced antibodies, whereas a T cell immune response is needed for disease attenuation. Hence, a robust understanding of B and T cell function is needed for proper immunization (9).

Multiple determinants modulate the primary vaccine antibody response in healthy individuals; they include the vaccine type, live versus inactivated, protein versus polysaccharide, and use of adjuvants (10). They also include the nature of the antigen and its intrinsic immunogenicity (11), the dose of the antigen, the route of administration, the vaccine schedule, and the age at administration (12). In addition, genes play a direct role in the body’s response to vaccination even in healthy individuals (13, 14). For each of the above determinants, there might be a particular mechanism involved and is further influenced by other factors including extremes of life, acute or chronic diseases, immunosuppression, and nutrition status (12).

Early life immune responses are limited by (1) limited magnitude of antibody responses to polysaccharides and proteins, (2) short persistence of antibody responses to protein, (3) influence of maternal antibodies, and (4) limited CD8+ T cell and interferon-gamma responses. Such factors are difficult to study in human infants due to neonatal immune immaturity and the inhibitory influence of maternal antibodies, which increase with gestational age and wane a few months post-natal (7).

On the other hand, in elderly persons, the immune system undergoes characteristic changes, termed immunosenescence, which leads to increased incidence and severity of infectious diseases and to insufficient protection following vaccination (15). Vaccines induce both innate (non-specific) and adaptive (specific) immune responses, which decline substantially with age thus leading to the decreased efficacy of vaccines in elderly persons. In the elderly, the innate immune response will witness a reduced phagocytic capacity of neutrophils and macrophages, a decrease in their oxidative burst, and impairment in the up-regulation of MHC class II expression among other parameters (16). In addition, persistent inflammatory processes occur with increasing age and may reduce the capacity to recognize stimuli induced by pathogens or vaccines. For the elderly, improved special antigen delivery systems are needed to overcome these limitations (12).

Furthermore, the adaptive immune response is functionally defective in the elderly. The involution of the thymus with aging leads to a decrease in content and in output of mature naïve T cells into the periphery, which hampers the induction of adaptive immune responses to neoantigens. In the context of primary vaccination, this causes reduced response rate (7–12). B cells also undergo age-related changes that aggravate the functionality of B cells colonies. As effector B cells accumulate, naïve B cells decrease in number and this leads to a reduction in the diversity of antibody responses. In brief, vaccines tailored to the needs of the elderly will have to be developed, taking into consideration these limitations in order to improve protection in this population.

## Vaccine Efficacy and Effectiveness in the Context of the Translational Research Map

In 2010, Weinberg and Szilagyl eloquently approached the issues of efficacy and effectiveness clarifying the road to correctly answer the relevant but complex question: “How well does the candidate vaccine prevent the disease for which it was developed?” They highlighted clearly the distinction between efficacy (individual level) and effectiveness (population level), which are often confused terms that fit well into the new paradigm of translational research (15). At about the same time, Curns et al. elaborated on the distinction between the epidemiologic concepts of vaccine efficacy and effectiveness within the context of translational research (17). Such concepts were also addressed earlier, but slightly differently, by Clemens and co-workers in two separate publications in 1984 and 1996, and also by Orenstein et al. in 1989 (18–20).

Accordingly, vaccine efficacy is measured as the proportionate reduction in disease attack rate when comparing vaccinated and unvaccinated populations. Vaccine efficacy studies always have rigorous control for biases through randomized prospective studies and vigilant monitoring for attack rates (15). In addition to proportionate reduction in attack rates, these studies can furthermore assess outcomes through hospitalization rates, medical visits, and costs. Despite the complexity and expenses that arise from the initial trials, they are needed to establish vaccine efficacy (15).

On the other hand, the related but distinct concept of vaccine effectiveness has always been compared to a “real world” view of how a vaccine reduces disease in a population. As such, it can evaluate risks versus benefits behind a vaccination program under more natural field conditions rather than in a controlled clinical trial. Vaccination program efficiency is proportional to vaccine potency or efficacy in addition to the degree and success of immunization of the target groups in the population. In brief, it is influenced by other non-vaccine-related factors that could influence the outcome. The “real world” picture provided by vaccine effectiveness data is desirable in planning public health initiatives, an advantage that makes these studies attractive. Translating research data into real public health application are a process that has been reengineered by the NIH as part of a road map for future research. Consequently, a new expanded definition of translational research, consisting of four steps was proposed, which fits nicely within the continuum of vaccine research (21). In this new process of phase I to phase IV clinical trials, safety, immunogenicity, efficacy, and post-licensure effectiveness of a particular vaccine are assessed ending up in phase IV with the burden of the disease (15).

Vaccines stood the test of time and many techniques have been introduced into the world of vaccination. Practitioners used to write articles about their vaccinating instruments and techniques. According to John Kirkup, vaccinators and physicians used various instruments and techniques to inject the vaccinating material into the human body. More than 45 different vaccinating instruments have been recorded in British, American, German, and French catalogs between the years 1866 and 1920; most of them are out of use nowadays (22).

## Beginning of Vaccines

There are multiple major landmarks in the history of vaccines. It was reported that the origin goes as far back as Hippocrates, the father of modern medicine, 400 B.C. He described mumps, diphtheria, and epidemic jaundice among other conditions (23). The earliest methods of immunization and protection against smallpox date back to about 1000 A.D., and are attributed to the Chinese. It has been said that the son of a Chinese statesman was inoculated against smallpox by blowing powdered smallpox sores into his nostrils (24). Another method used for inoculation was the removal of fluid from the pustules of an infected individual and subsequently rubbing it into a skin scratch of a healthy individual. This procedure was later introduced into Turkey around 1672, long before reaching Europe (25). It took six centuries for variolation to be introduced to Great Britain, in 1721 (26).

## Through the Eighteenth Century

The eighteenth century was marked by several major events that started with the spread of variolation from Turkey and China to England and America, followed, in the late eighteenth century, by Edward Jenner’s breakthrough of vaccination.

## Variolation from Turkey to England

Variolation, derived from the Latin word *varus*, meaning “mark on the skin,” or inoculation, derived from the Latin word *inoculare*, meaning “to graft,” are two words that were used interchangeably in describing the aforementioned immunization process. By 1715, variolation was introduced to England after the pursuit of an English aristocrat, Lady Mary Wortley Montague, who had been personally inflicted with an episode of smallpox. After being informed of the method of variolation, she made the embassy surgeon, Charles Maitland, perform the procedure on her 5-year-old son in 1718 in Turkey. In 1721, Dr. Charles Maitland performed the first English variolation on Lady Montague’s 4-year-old daughter after their return to London (27).

Lady Montague became a great proponent of the procedure and worked thoroughly on advocating this process for its ability to protect against the spread of smallpox.

Data from the U.S. National Library of Medicine and the NIH showed that 1–2% of those variolated died as compared to 30% of those who contracted the disease naturally. Correspondingly, Rev. Cotton Mather and Dr. Zabdiel Boylston introduced variolation in America and were also great advocates of this procedure especially since, in the same year, there was a smallpox epidemic in Boston that killed hundreds (28). However, Lady Montague, Rev. Mather, and Dr. Boylston faced great opposition regarding their promotion of variolation even with the presentation of the comparative analysis of fatality rates, which reached 2% for those variolated compared to 14% for the naturally occurring disease (27).

## Spreading the Word

Despite some variolation-related deaths, the word of inoculation kept spreading along with data suggesting that variolation was still the safeguard against the spread of smallpox. In addition, Benjamin Franklin, who lost his son in 1736, wrote: “I long regretted that I had not given it to him by inoculation, which I mention for the sake of parents who omit that operation on the supposition that they should never forgive themselves if a child died under it; my example showing that the regret may be the same either way, and that therefore the safer should be chosen” (24). In 1759, Dr. William Heberden, at his own expense and with the support of Benjamin Franklin, wrote a pamphlet entitled “Some Account of the Success of Inoculation for the Small-Pox in England and America: together with plain instructions by which any person may be enabled to perform the operation and conduct the patient through the distemper” (29).

## Edward Jenner’s Breakthrough

Toward the late eighteenth century came Jenner’s breakthrough in finding a safer immunizing technique than variolation, which is vaccination.

The method of variolation had low yet significant death rates; therefore, physicians were on the quest of finding a new and more secure method of immunization with minimal or no death rates. On this basis, an English physician named Edward Jenner (1748–1823) searched for a cure for smallpox, a debilitating disease that rendered the world helpless. Jenner became interested in certain individuals who were immune to smallpox because they had contracted cowpox in the past. He personally witnessed this when he learned of a dairymaid that was immune to smallpox due to her previous infection with the cowpox virus, usually transmitted from infected cattle. During that time, an English farmer named Benjamin Jesty personally took charge of inoculating his wife and children with fresh matter from a cowpox lesion in one of his cows out of fear of having his wife and children become victims of the smallpox epidemic. He applied this method after having contracted cowpox himself and believing he was immune to smallpox. He never published his results even though his wife and children did not show symptoms after being exposed to smallpox (27). During these years, there were still outbreaks of smallpox. George Washington, after surviving smallpox, ordered mandatory inoculation for his troops in 1777 (27).

After many speculations on the role of cowpox and its immunizing effect against smallpox, Jenner, in 1796, inoculated an 8-year-old boy named James Phipps using matter from a fresh cowpox lesion on the hands of a dairymaid named Sarah Nelms who caught them from her infected cattle. After several days, Jenner inoculated the boy again but this time with fresh matter from a smallpox lesion and noted that the boy did not acquire the disease proving that he was completely protected (27). A few years later, word of his success circulated among the public, and Jenner wrote “*An Inquiry into the Causes and Effects of the Variolae Vaccinae, a Disease Discovered in some of the Western Counties of England, particularly Gloucestershire and Known by the Name of CowPox*,” after adding several cases to his initial achievement with the boy Phipps. At first, his publication and achievement did not stir any interest in his community, but with time, word of Jenner’s breakthrough began spreading (27).

The late eighteenth century was characterized by the implementation of the new process of immunization, vaccination, which required the inoculation of fresh matter from cowpox lesions into the skin of healthy individuals.

## Through the Nineteenth Century

The nineteenth century was a major landmark in the history of vaccines since it witnessed discoveries made by Louis Pasteur, the father of microbiology, and Robert Koch, the scientist who discovered the germ responsible for tuberculosis (26).

## Vaccination Versus Variolation

In the beginning of the nineteenth century, the term “Vaccination” was introduced by Richard Dunning from the Latin word for cow “Vacca.” After becoming aware of the fact that vaccination was more secure than variolation, several physicians initiated movements against the use of variolation and advocated for its eradication. Dr. Jean de Carro, for example, aided in the elimination of variolation and its substitution with vaccination. Some of the major efforts implemented in America were initiated by Dr. Benjamin Waterhouse, who received the vaccine from Edward Jenner and vaccinated his own family. He later proved that they acquired immunity when they remained asymptomatic after he infected them with smallpox. Waterhouse worked effectively on making vaccination universal in the U.S. Unfortunately, like any other medical breakthrough, problems arose both because Waterhouse aimed at making profit and the public was not ready to implement these procedures. However, after breaking his initial monopoly, Waterhouse accepted to share his vaccines and made the supplies available to other physicians (24). Despite all these efforts, smallpox epidemics continued to occur and Jenner stated in a pamphlet that he wrote, “The annihilation of the small pox, the most dreadful scourge of the human species, must be the final result of this practice.” Eradication was finally achieved 176 years later. The time it took could be attributed to the fact that Jenner did not think of the necessity of revaccination nor of the instability of vaccines, which made them unable to handle different environmental conditions, including countries other than England (30).

The late nineteenth century was distinguished by Pasteur’s achievements that made him the father of vaccines after creating the first laboratory vaccine. Louis Pasteur (1822–1895), a French chemist and microbiologist, was the first to propose the “Germ Theory” of disease in addition to discovering the foundations of vaccination (26). He studied chicken cholera and received strains of bacteria causing anthrax and septic *Vibrio*. Pasteur started his experiments by intentionally infecting chickens by feeding them cholera-polluted meals and then recording the fatal progression of the illness. At first, Pasteur was using fresh cultures of the bacteria to inoculate the chickens, most of which did not survive. During that time, Pasteur had to go on a holiday, so he placed his assistant in charge of injecting the chickens with fresh cultures. However, his assistant accidentally forgot to perform the injections, and the bacterial cultures were left in a medium that was exposed to room air for about a month. Later, the attendant injected the chickens with the now “attenuated” strain of bacteria resulting in mild, non-fatal symptoms. Pasteur later re-injected these chickens, but this time with fresh bacteria. To his surprise, they did not get ill. Ultimately, Pasteur reasoned that what made the bacteria less deadly was exposure to air, mainly oxygen. Pasteur used the French verb “vacciner” during the years 1879 and 1880 to describe how he was able to provide total body immunity through vaccination by inoculation of an attenuated virulence which was the first vaccine made by a human in the laboratory (31).

Pasteur also developed the anthrax vaccine in his laboratory, not long after performing his studies on chicken cholera. In 1881, Pasteur used his own anthrax vaccine, which contained attenuated live bacterial cultures in addition to carbolic acid, and demonstrated that all vaccinated animals survived while the control group died (32). During the same year, Louis Pasteur in France and George Miller Sternberg in the U.S. almost simultaneously and independently isolated and grew the pneumococcus organism. Later in 1884, Pasteur successfully fought rabies that was endangering the European livestock by using his attenuated rabies vaccine obtained from desiccated brain tissue inactivated with formaldehyde, which provided immunity to dogs against rabies in his experiments (26). He reported his success to the Academy of Sciences in France, and a year later, he applied his original vaccine 60 h after a 9-year-old boy was bitten several times by a rabid dog. The boy survived after being first inoculated with the most attenuated organisms, then subsequently with less attenuated organisms each day for 10 days (33). In 1888, the Pasteur Institute was established as a rabies treatment center as well as an infectious diseases research and training institute.

## From Live Vaccines to Killed Vaccines

After Pasteur’s successful live vaccines, a new type of vaccine was introduced in the last few years of the nineteenth century. These were killed vaccines, which were directed against three chief bacterial causes of human morbidity: cholera, typhoid, and the plague. The first cholera vaccine used to immunize humans was actually a live vaccine developed by Jaime Ferran (1852–1929), which provided a high level of protection during the 1884 epidemic in Spain. However, the first killed vaccine for cholera was developed in 1896 by Wilhelm Kolle (1868–1935) and was used in Japan in 1902 with over 80% efficiency. The credit for developing the killed typhoid vaccine during the 1890s goes to both Richard Pfeiffer and Almroth Wright who made great contributions. Wright was later credited for carrying out the “first large-scale vaccination using a killed typhoid vaccine” (34). Finally, the killed vaccine for plague was first developed in 1896 by Haffkine, who was one of Pasteur’s followers, when an epidemic struck Bombay.

## Late Nineteenth and Twentieth Century

During this period, vaccine production was taken over by factory-type laboratories, which formed the precursors of the biological products supply houses. Many types were produced.

## Toxoid

Paul Ehrlich (1854–1915), a German physician and scientist who worked under a contractual collaboration with Behring, noted the existence of toxoids in the late 1890s. He also promoted enrichment and standardization protocols. These protocols enabled the exact determination of quality of the diphtheria antitoxins. In 1907, it was demonstrated that toxoids could be used to durably immunize guinea pigs. It is crucial to briefly address the historical background of the bacterial infections that led to some of the earliest and most successful use of toxoids, inactivated forms of bacterial toxins, for the purpose of immunization. Until the twentieth century, diphtheria, tetanus, and pertussis proved to be significant causes of illness and death with no effective treatments or prevention in sight. Fortunately, advances in 1890 improved the prognosis of numerous future patients (35). At the end of the nineteenth century, especially in 1896 and 1897, the cholera and typhoid vaccines were developed, followed by the introduction of the plague vaccine. The latter was preceded by the preparation of anti plague horse serum at the Pasteur Institute by Alexandre Yersin.

Yersin demonstrated disease protection in animals. Later, he went to China to try his vaccine on humans during a plague epidemic (26).

## Diphtheria

Diphtheria is a potentially fatal disease that primarily involves tissues of the upper respiratory tract and kills its victims slowly by suffocation. In 1884, a German physician, Edwin Klebs (1834–1913), was able to successfully isolate the bacteria that proved to be the etiological agent of the disease. It was later proved that toxin production is initiated only after the bacteria are themselves infected by a specific virus or a bacteriophage carrying the toxin’s genetic instructions (35).

In France, during the year 1888, Emile Roux discovered the diphtheria toxin. His discovery led to the development of passive serum therapies through the scientific contributions of many, including Emil Von Behring and Paul Ehrlich (26). Similarly, the etiological agent of Pertussis, commonly known as the “whooping cough,” was found to be a bacterium isolated from infected patient tissues in 1906 (36). Tetanus was similarly a significant cause of mortality usually resulting from dysfunction of the autonomic nervous system or the respiratory muscles (37). In 1884, another German scientist, Arthur Nicolaier (1862–1942), correlated tetanus with an anaerobic soil bacterium found in wounds. A few years later, the Japanese investigator Shibasaburo Kitasato (1853–1931) was able to isolate this bacterium (35). At the beginning of World War I in 1914, the tetanus toxoid was introduced following the development of an effective therapeutic serum against tetanus by Emil Von Behring and Shibasaburo Kitasato. The rabies and typhoid vaccines were then licensed in the U.S. as the etiology of these destructive diseases was slowly being uncovered, by Shibasaburo Kitasato along with Emil von Behring (26). They discovered that the serum of animals that had been exposed to sub-lethal doses of the bacteria involved in tetanus and diphtheria was protective against the lethal effects associated with these pathogens by having an antitoxin effect when injected into another animal. Additionally, this discovery, which earned Behring the inaugural Nobel Prize for Physiology and Medicine in 1901, was the concept of passive transfer in addition to serum therapy. He proved that serum could be acquired from immune animals and transferred to others as protection (38). Once this concept made its way to clinical practice in 1891, technical problems were faced while developing the right antitoxin concentration and potency. As a result, in the early twentieth century, the U.S. Congress enacted the Biologics Control Act legislation “to regulate the sale of viruses, serums, toxins, and similar products” to ensure medication quality control. Nevertheless, with the increasing use and popularity of antitoxins derived from animal serum, scientists began to observe a syndrome now called serum sickness, or a reaction to immune-complexes formed from combining high concentrations of antigens with antibodies. This eventually led to the use of human rather than animal serum in order to decrease the frequency of adverse events; still, serum therapy was not perfect in preventing disease due to the frequency of adverse events and its brief duration of action. Later on, combining diphtheria toxin and antitoxin in the same syringe proved much more effective in decreasing mortality rate. This combination became commercially available in 1897. This was the first step in the shift from passive to active immunization (35). In 1923, Gaston Ramon (1886–1963), a French veterinarian working at the Pasteur Institute, used a diphtheria toxoid produced by formalin and heat inactivation without the use of antitoxin to safely induce active immunity in humans. This product, termed anatoxine, was the basis for the novel and clinically effective toxoid vaccine against diphtheria. Experiments followed to improve the durability of the protective response of the vaccine, and in 1926, the importance of aluminum salts as an adjuvant added to the vaccine to augment the immune response to the antigen, became apparent (38). This was discovered by Alexander Thomas Glenny (1882–1965) who proved that toxoid alone produced a lower level of antibody and immunity than desired, whereas better immunity was achieved when an inflammatory reaction was triggered. With these significant improvements, tetanus and diphtheria toxoids became routinely used across America and Europe in the 1930s and 1940s (35).

Since then, refinements have been made to these vaccines to yield higher purity and reduce the number of booster doses. Nowadays, widespread childhood vaccination is reducing the burden of these diseases. While this is a huge advantage, vaccines may potentially produce adverse effects that can discourage their acceptance by some populations. This has led to numerous safety movements which culminated in the congressionally legislated National Childhood Vaccine Injury Act in the 1980s created to compensate families for selected adverse events potentially related to mandatory childhood vaccinations (37). Nevertheless, global recommendations continue to call for routine immunization of children against diphtheria, tetanus, and pertussis with the combined DTP vaccine to sustain immunity in childhood and adolescence. DTP has, therefore, become one of the most widely used vaccines to achieve widespread immunity across age groups (35).

## Tuberculosis and BCG

Tuberculosis, otherwise known as the “Great White Plague,” is another disease that started spreading as an epidemic once industrialization began. This disease caused approximately 15% of deaths in the eighteenth and nineteenth centuries across all socioeconomic groups (39).

A French physician named Jean Antoine Villemin (1827–1892) demonstrated that the mode of transmission of disease is through the respiratory system. Robert Koch (1843–1910), known as the founder of modern bacteriology, revealed in 1882 that the causative agent of the disease is *Mycobacterium tuberculosis*, which later became known as Koch’s bacillus (40). Following this discovery, Koch created what later came to be known as Koch’s postulates, which listed the criteria necessary for proof of bacterial causality: “the organism must be present in diseased tissues; it must be isolated and grown in pure culture; and the cultured organisms must induce the disease when inoculated into healthy experimental animals” (39).

In 1908, two bacteriologists working in the Pasteur Institute in Lille, Albert Calmette (1863–1933) and Camile Guerin (1872–1961), announced their discovery of *Mycobacterium bovis*, which is a strain of tubercle bacilli that could be used to create a vaccine against tuberculosis. This occurred after it became evident that different forms of the bacterium were required to prevent or treat tuberculosis, including non-pathogenic, attenuated, or killed tubercle bacilli from different sources, including human, bovine, and equine. This strain had an attenuated virulence while maintaining its antigenicity and became known as BCG (40).

Bacille Calmette–Guerin vaccinations proved to be successful in animal studies in 1921 and were soon used as an oral vaccine to immunize humans against tuberculosis. In 1927, the BCG vaccine, constituted by the live-attenuated *M. bovis*, was first used in newborns. It has become the most widely administered of all vaccines in the WHO Expanded Program for Immunization, but has been estimated to prevent only 5% of all potentially vaccine-preventable deaths due to tuberculosis (26). Despite its imperfections, BCG remains the only effective vaccination for protection against human tuberculosis (39).

## Yellow Fever

Yellow fever is a highly fatal infection caused by a small, enveloped, single-stranded RNA virus and results in renal, hepatic, and myocardial injury, along with hemorrhage and shock (41). Unlike previously mentioned diseases, the history of yellow fever is highly uncertain and filled with misconceptions. Early work on immunization against the disease began with Carlos Finlay (1833–1915) in the 1870s and 1880s when Koch’s postulates were becoming increasingly accepted. Finlay proposed that mosquitos carried the yellow fever “germ.” He attempted to prove it by feeding mosquitos that had fed on yellow fever patients. However, it was later revealed that his process failed due to the lack of an incubation period within the mosquito, which is a transmission requirement that Finlay was unaware of (42).

Since 1900, significant advances have been made in creating a vaccine by the Yellow Fever Commission, which was originally led by Walter Reed (1851–1902) along with Jesse Lazear, Aristedes Agramonte, and James Carroll. Reed’s experiments took Finlay’s discovery one step further by adding an incubation period of approximately 2 weeks and achieved the same positive results. When mosquitos bite non-immune individuals after feeding on individuals who had yellow fever, none of the non-immune subjects died and very few suffered disease. This led the Commission of investigators to a major discovery, namely, the identification of the Asibi strain, which is the parent strain of the present 17D vaccine, obtained via continuous indirect passage through the *Aegypti* mosquitos and direct passage through monkeys. In addition to identifying the etiological agent of the disease, the Commission also identified rhesus monkeys as susceptible hosts, hence providing a means for testing future vaccine attempts. This paved the way for Max Theiler and other Rockefeller Foundation scientists to develop a successful live-attenuated vaccine for yellow fever in 1937. “The most important experimental passage series – designated 17D – used a virus that had been subcultured eighteen times in whole mouse embryos, followed by 58 passages in whole-minced chick embryo cultures, after which the virus was passed in minced chick embryo depleted of nervous tissue.” Theiler himself was actually one of the first individuals to be successfully vaccinated. The vaccine was quickly implemented, and alternative vaccines shown to be more dangerous were discontinued (42).

## Influenza

Influenza has proved to be very difficult to trace back in history due to its non-specific symptoms and features. It was not until the early twentieth century that influenza outbreaks began to be systematically studied due to well-documented clinical descriptions and epidemiological data. In 1918, the “Spanish flu” influenza pandemic was responsible for 25–50 million deaths worldwide and more than one-half million in the U.S. This virus was unusual because it spread so quickly, was so deadly (26). Richard E. Shope (1901–1966), a physician who conducted his research in the Department of Animal Pathology at The Rockefeller Institute in Princeton, was the first to isolate influenza virus; a member of the orthomyxovirus family, from a mammalian host in 1931 (43). He was able to induce the syndrome of swine influenza in pigs by applying respiratory secretions intranasally. He also isolated a bacterium from the respiratory tract of infected pigs called *Haemophilus influenzae suis*. When this bacterium was combined with a filterable agent and inoculated, the pigs developed the clinical manifestations of swine influenza. These two agents seemed to act synergistically with the virus to damage the respiratory tract hence creating the suitable environment needed for the virus to exercise its pathological effects. In 1933, scientists from the British National Institute for Medical Research including Christopher Andrews, Wilson Smith, and Patrick Laidlaw successfully isolated and transmitted the influenza virus from humans. Throughout this year, “Burnet has successfully cultivated the organism in chick embryos; other influenza types had been recognized; neutralizing antibodies had been identified and quantitated; and viral surface glycoproteins, H and N had been described” (43).

These discoveries led scientists to introduce the inactivated vaccine in the mid-1940s that is still used to this day (44). The influenza A/B vaccine was initially presented to the Armed Forces Epidemiological Board in 1942. It was licensed following the war and used for civilians in 1945 in the U.S. Starting 1985, a series of vaccines were licensed for *Haemophilus influenza* type b (Hib) polysaccharide vaccines. These vaccines are recommended routinely for children at 15 and 24 months of age. The vaccine was, however, not consistently immunogenic in children <18 months of age. In 1987, the protein-conjugated Hib vaccine was licensed and in the next 2 years, it became available. During 1996, a combined vaccine Hib conjugate and Hepatitis B was licensed. Later on, in 2003, the first nasally administered influenza vaccine was licensed. This live influenza A and B virus vaccine was indicated for healthy, non-pregnant persons ages 5–49 years. The contracts to develop vaccine against the H5N1 avian influenza virus were awarded to Aventis Pasteur and to Chiron in 2004. During the following year, an inactivated, injectable influenza vaccine was licensed. It was indicated for adults 18 years of age and older.

During the same year, the FDA approved Afluria, a new inactivated influenza vaccine, for use in people aged 18 years and older. Two years later in 2009, the Department of Health and Human Services, supported the building of a facility to manufacture cell-based influenza vaccine. It also directed toward development of a vaccine for novel influenza A (H1N1). During the same year, the FDA approved four vaccines against the H1N1 influenza virus high-dose inactivated influenza vaccine (Fluzone High-Dose) for people aged 65 years and older. In 2012, the FDA approved several vaccines: HibMenCY a new combination of meningococcal and Hib vaccine for infants; Flucelvax, which is the first seasonal influenza vaccine, manufactured using cell culture technology and a quadrivalent formulation of Fluarix (26).

Unfortunately, one of the difficulties in dealing with influenza is the continuous mutability of the viral genome necessitating annual reassessments and reformulations of the vaccine. This has led to a suboptimal effectiveness of influenza vaccines, which are only successful against strains included in the vaccine formulation or strains of homogenous subtype. Several pandemics were caused by the influenza virus: during the years 1957–1958, The “Asian” influenza pandemic caused by H2N2 influenza virus resulted in an estimated 70,000 deaths in the U.S. alone and in the years 1968–1969, the “Hong Kong” influenza pandemic caused by an H3N2 influenza virus induced roughly 34,000 deaths in the U.S. (26). Future studies should focus on producing vaccines protective against variant strains and creating surveillance systems to detect novel strains in time to formulate the proper vaccines.

## Poliomyelitis

Poliomyelitis, or Polio, is an intestinal infection spread between humans through the fecal–oral route. It is a disease of the developed nations striking younger individuals most frequently in warmer weather. One of the most famous polio victims, President Franklin D. Roosevelt, founded the National Foundation for Infantile Paralysis in 1938, later known as the March of Dimes (26). It is well established that better hygiene decreases childhood exposure to the disease, when infection would usually be milder since protective maternal antibodies are present (45). In 1954, the Nobel Prize in Medicine was awarded to John Enders, Thomas Weller, and Fredrick Robbins for their discovery of the ability of poliomyelitis viruses to grow in tissue cultures (26). Two major lifelong competitors were involved in the race for the Polio vaccine, Jonas Salk (1914–1995) and Albert Sabin (1906–1993). Salk took a more traditional route using a killed-virus approach, which did not involve natural infection in acquiring immunity. Instead, his approach involved a fully inactivated virus that still had the ability to induce protective antibodies. Sabin, on the other hand, set out to create a live-virus vaccine based on the belief that this would trigger natural immunity and provide a lasting protection. Salk had speed, simplicity, and safety on his side since a killed-virus did not have the ability to revert to virulence, whereas the live-virus vaccine could be given orally, establish longer lasting immunity, and offer passive vaccination through the excreted weakened virus potentially immunizing a large portion of non-vaccinated communities (46).

Not surprisingly, Salk’s vaccine was the first to make it to the population. Following successful clinical trials in 1954, six companies began mass production of the vaccine. Unfortunately, Salk’s vaccines were soon suspended and recalled when contaminated samples were found in the market due to poor monitoring and control in some laboratories leading to serious health consequences and national panic. The first Cutter polio vaccine incident was reported on April 25, 1955 with 5 more cases reported just a day later with the number eventually rising to 94 of those vaccinated and in 166 of their close contacts. On April 27, the Laboratory of Biologics Control requested that Cutter Laboratories recall all vaccines and the company did so immediately. On May 7, the Surgeon General recommended that all polio vaccinations be suspended pending inspection of each manufacturing facility and thorough review of the procedures for testing vaccine safety. The investigation found that live polio virus had survived in two batches of vaccines produced by Cutter Laboratories. Large-scale polio vaccinations resumed in the fall of 1955 (26). At the same time, Sabin had been making great advances with his live-virus vaccine since 1951. After successful clinical trials conducted in the Soviet Union that left polio virtually wiped out with no safety issues, it soon became the vaccine of choice in the West. The Polio Vaccination Assistance Act was enacted by Congress and was the first federal involvement in immunization activities. It allowed Congress to appropriate funds to the Communicable Diseases Center [later the Centers for Disease Control and Prevention (CDC)] to help states and local communities acquire and administer vaccines. At the beginning of the 1960s, the oral polio vaccine types 1, 2, and 3 as well as the trivalent product were licensed in the U.S. The first 2 were developed by Sabin and grown in monkey kidney cell culture, while the trivalent oral polio was developed to improve upon the killed Salk vaccine (26). As a result, in the late 1990s, the CDC recommended switching back to Salk’s killed-virus polio vaccine, while the WHO also advocated the switch for polio-free nations and the continued use of the favored live-virus vaccine for routine immunization (45).

The last two cases of wild type polio were reported in an unvaccinated Amish in 1979 and in a 5-year-old boy from Peru in 1991 (26). In 1990, the enhanced-potency inactivated poliovirus vaccine was licensed.

## Measles, Mumps, and Rubella

Following successful developments in the polio vaccine, attention soon shifted to three other common viral diseases of childhood: measles, mumps, and rubella.

The measles virus is an RNA virus from the genus *Morbillivirus* belonging to the Paramyxooviridae family. It causes an acute illness that includes fever, cough, malaise, coryza, and conjunctivitis, in addition to a maculopapular rash. In general, measles is a mild disease but, like many others, has the potential to cause serious complications. In addition, measles is known to be one of the most contagious human diseases causing major outbreaks to occur very often. Until the year 2000, measles was still the leading cause of vaccine-preventable childhood deaths worldwide (47).

John Enders (1897–1985), known as the “Father of Modern Vaccines” had a particular interest in revealing the virus responsible for measles. He isolated the Edmonston strain of the virus in 1954, which was named after the child from whom it was isolated. A formalin-inactivated measles virus vaccine derived from this strain was subsequently licensed in the U.S. in 1963. However, following the discontinuation of this vaccine in 1967 due to short-lived and incomplete immunity, over 20 further attenuated vaccines were developed and used throughout the world, most of which were also derived from the Edmonston strain (48). The first live-virus measles vaccine, Rubeovax, was licensed in 1963. Other live-attenuated virus measles vaccines were eventually licensed in the U.S. in 1965. The recommended age for routine administration was changed from 9 to 12 months of age.

The first national measles vaccine campaign was launched in 1966. The world recorded a 90% decreased incidence compared to the pre-vaccination years. In 1968, a second live, further attenuated measles virus vaccine was also licensed. In 1989, both the Advisory Committee on Immunization Practices (ACIP) and the American Academy of Pediatrics (AAP) issued recommendations for a routine second dose of the measles vaccine. During the mid-to-late 1980s, a high proportion of reported measles cases were in school-aged children (5–19 years) who had been appropriately vaccinated. These vaccine failures led to new national recommendations of a second dose of measles-containing vaccine (26).

Mumps is another acute viral illness. It is the only virus known to cause epidemic parotiditis in humans accompanied by fever, anorexia, headache, and malaise. K. Habel and John Enders isolated the virus in 1945 (26), and trials of formalin-inactivated mumps vaccine in humans began the same year by Joseph Stokes and colleagues and by Enders. This approach was abandoned in the 1950s due to short-lived immunity, and work began to develop live-attenuated mumps vaccines in 1959 by the vaccinologist Maurice Hilleman (1919–2005) and colleagues (48). Hilleman isolated the wild type virus from his daughter, Jeryl Lynn, who contracted the virus at the age of 5 and was recovering from it. It became known as the Jeryl Lynn strain of mumps virus. The mumps live-virus vaccine was licensed in December 28, 1967 (26). Trials with this attenuated virus resulted in 100% protective efficacy and the vaccine was licensed in the U.S. in 1967. This strain is still used to produce mumps vaccines until this day. It is given as part of the measles, mumps, and rubella (MMR) vaccine (49).

Rubella is a rash disease in children and adolescents caused by a filterable virus. It poses a severe threat to pregnant women and their children by potentially causing congenital deafness and cataracts. In 1964, a rubella epidemic swept the U.S. resulting in 12.5 million cases of rubella infection, with an estimated 20,000 newborns having congenital rubella syndrome (CRS), along with fetal and neonatal deaths in the thousands (26). The rubella virus was detected and isolated by two groups of scientists, Thomas Weller and Franklin Neva at Harvard Medical School, in addition to Paul Parkman and colleagues at the Walter Reed Army Institute of Research (WRAIR). Similar to measles and mumps, inactivated whole virus vaccines proved ineffective, so efforts turned to discovering a live-attenuated vaccine (26).

In 1963, Paul Parkman left WRAIR and joined Harry Meyer Jr. at the NIH Division of Biological Standards, and the pair developed the first live-attenuated rubella vaccine in 1966, HPV-77, which was subsequently included in the initial MMR vaccine used in the U.S. in the 1970s (26). Maurice Hilleman discovered the superior RA 27/3 vaccine that became the only vaccine used outside of Japan starting in the late 1970s. This vaccine maintained its preference due to many factors including increased durability and harmlessness to fetuses of inadvertently vaccinated pregnant women (47). In 1969, three rubella virus strains were licensed in the U.S.: HPV-77 strain grown in dog-kidney culture, HPV-77 grown in duck-embryo culture, and Cendehill strain grown in rabbit-kidney culture. A decade later, in 1979, the RA 27/3 (human diploid fibroblast) strain of rubella vaccine (Meruvax II) by Merck was licensed. All other strains were discontinued. Merck’s combined trivalent MMR as well as the combined measles and rubella vaccine (M-RVax) developed by Maurice Hilleman and colleagues, was licensed by the U.S. government in 1971 (26), and is still in use today. Moreover, the age for routine vaccination with MMR vaccine was changed from 12 to 15 months in the year of 1976. The next vaccine that combined measles, mumps, rubella, and varicella antigens (Proquad) was licensed in 2005. It was indicated for use in children 12 months to 12 years. In response to the association of this vaccine with autism, in 2004, the eighth and final report of the Immunization Safety Review Committee was issued by the Institute of Medicine concluded that the body of epidemiological evidence favors rejection of a causal relationship between the MMR vaccine and autism (26). Combination vaccines hold many advantages including reduced need for several injections, therefore, reducing the incidence of vaccination site reaction (48).

## Hepatitis

The etiological agent of clinical hepatitis, identified by its distinguishing yellow jaundice, was found to be infectious in the early 1900s. The different hepatitis strains, A and B, were first differentiated in 1942 (26). In the mid-1960s, Blumberg and co-workers and Prince discovered hepatitis B surface antigen in the circulating blood of carriers of the infection. Deinhardt et al. soon followed this discovery with that of the hepatitis A virus (49). Provost et al. successfully prepared a killed hepatitis A vaccine in 1986, which proved to be safe and highly effective in extensive clinical trials. The first inactivated hepatitis A vaccine (Havrix) was licensed in 1995. The following year, a second inactivated vaccine (Vaqta) also became available (50).

Hepatitis B, on the other hand, rarely causes any severe risk as a primary infection. However, those who develop a chronic persistent infection may continue to have severe disease for the rest of their lives. This may even lead to cirrhotic destruction of the liver due to host immune response to the virus. The discovery of the surface antigen particles of the hepatitis B virus by Blumberg and colleagues in the plasma of human carriers was followed by attempts to create a vaccine. In 1968, a killed hepatitis B vaccine was developed and clinical trials began in 1975 proving the safety and efficacy of the vaccine. Merck and Pasteur Institute subsequently independently licensed the plasma-derived vaccine in 1981 (50). On July 23rd 1986, the recombinant hepatitis B vaccine (Recombivax HB) was licensed. Using recombinant DNA technology, Merck scientists developed a hepatitis B surface antigen subunit vaccine. Three years later, on August 28th 1989, the recombinant hepatitis B vaccine (Engerix-B) was licensed. A decade later in 1999, the FDA approved a two-dose schedule of hepatitis B vaccination for adolescents 11–15 years of age using Recombivax HB (by Merck) with the 10-μg (adult) dose at 0 and 4–6 months later. At the beginning of the new millennium, in 2001, a combined hepatitis A inactivated and hepatitis B (recombinant) vaccine, Twinrix was licensed. The following year, a vaccine combining diphtheria, tetanus, acellular pertussis, inactivated polio, and hepatitis B antigens (Pediarix) was licensed (26). In conclusion, fortunately, both hepatitis A and B are now preventable due to the discovery of these highly effective vaccines that proved to maintain long-term immunity in vaccinated individuals (50).

## MID Twentieth and Twenty-First Century

In 1966, the World Health Assembly called for global smallpox eradication, which was launched the following year. During the first year of the program 217,218 cases of polio were reported in 31 countries that were endemic to smallpox. Four years later, the CDC recommended discontinuation of routine vaccination for smallpox in the U.S. following a greatly reduced risk of disease (26).

During the 70s, especially in 1974, the Expanded Program on Immunization was created within WHO, in response to poor immunization levels in developing countries (<5% of children in 1974). The following vaccines were used by the Expanded Program on Immunization: BCG, Polio, DTP, measles (often MMR vaccine), yellow fever (in endemic countries), and hepatitis B. Three years later, in October 1977, the last case of naturally acquired smallpox occurred in the Merca District of Somalia. In the same year, the first pneumococcal vaccine was licensed, containing 14 serotypes (of the 83 known serological groups) that composed 80% of all bacteremic pneumococcal infections in the U.S. (26).

On May 8 1980, the World Health Assembly declared the world free of naturally occurring smallpox. On the other hand, in July 1983, two enhanced pneumococcal polysaccharide vaccines (Pneumovax 23 and Pnu-imune 23) were certified. These vaccines included 23 purified capsular polysaccharide antigens of *Streptococcus pneumoniae* and replaced the 14-valent polysaccharide vaccine licensed in 1977. A few years later, in 1988, the World Health Assembly passed a resolution to eradicate polio by the year 2000 (26). Later on, in 1992, the Japanese encephalitis (JE) inactivated virus vaccine (JE-Vax) was licensed.

During the year 1994, The Expanded Program for Vaccine Development and the Vaccine Supply and Quality Program were merged creating the Global Program for Vaccines and Immunization. During the same year, the Western Hemisphere was finally labeled as “polio-free” by the International Commission for the Certification of Polio-Eradication.

The 1996 was another monumental year with the launching of the International AIDS Vaccine Initiative (IAVI) that called for the speedy development of a human immunodeficiency virus (HIV) vaccine for use worldwide. This in turn led to the introduction of the Scientific Blueprint for AIDS Vaccine Development. IAVI was funded by several NGOs and foundations. It is a Collaborating Center of the Joint United Nations Program on HIV/AIDS (UNAIDS) whose efforts led finally lead to the first possible vaccine against HIV (Aidsvax) which reached Phase III trials, the largest recorded human HIV vaccine trial at that time. The trial involved 5400 volunteers from the U.S., Canada, and the Netherlands, the majority of whom were men who have sex with men (26). Preliminary results from the trial of AIDS VAX (VaxGen) vaccine were reported in early 2003. While the vaccine was shown to be protective amongst non-Caucasian populations, especially African-Americans, the same effect was not reproducible in Caucasians (26).

During the same year, the Children’s Vaccine Program was established at WHO’s Program for Appropriate Technology in Health (PATH). The program’s goal was to provide vaccines to children in the developing world and to accelerate research and development of new vaccines. The first vaccines purchased were Hib, Hepatitis B, Rotavirus, and Pneumococcal, which were not commonly used in the developing world (26).

At the beginning of the new millennium, the Western Pacific Region of the world was certified as polio-free. During the next 2 years, the European Region also became certified as polio-free. In 2006, the FDA licensed the first vaccine developed to prevent cervical cancer (Gardesil), precancerous genital lesions and genital warts due to human papillomavirus (HPV) types 6, 11, 16, and 18. The first smallpox vaccine for certain immune-compromised populations was delivered under Project BioShield on July 10th 2010. The following year 2010, the WHO declared the “Decade of Vaccines” and in 2012, the United Nations Foundation launched Shot@Life campaign (26).

## Varicella Zoster: Herpes Virus

Varicella (“chickenpox”) is caused by the varicella zoster virus (VZV). Michiaki Takahashi, Professor of Virology at the Research Institute for Microbial Diseases at Osaka University, successfully produced the Oka vaccine strain of live, attenuated varicella vaccine in the 1970s. Takahashi was able to make this remarkable advance at a time when very few viruses had been attenuated to produce efficacious live-virus vaccines including yellow fever, polio, measles, mumps, and rubella as previously mentioned. The VZV vaccine is the first and only licensed live, attenuated herpesvirus vaccine in the world. Numerous trials in the early 1970s continued to prove the safety and efficacy of the vaccine in both healthy and immunocompromised, high-risk individuals. As a result of these successful trials, the live varicella virus vaccine (Varivax) was licensed in 1995 for the active immunization of persons 12 months of age and older (51). About 10 years later, in 2006, VariZIG, a new immune globulin product for post-exposure prophylaxis of varicella, became available under an Investigational New Drug Application Expanded Access Protocol (26).

As a herpesvirus, VZV possesses the unique ability to establish latent infection subsequent to primary infection. Zoster results from reactivation of latent VZV that spreads through nerves to the skin. Therefore, one fear associated with this vaccination was the possibility that it could increase the incidence and/or severity of zoster when compared to natural disease. Conversely, it was actually shown that following vaccination, zoster is less common than after natural infection (51). In 2006, the FDA licensed a new vaccine to reduce the risk of shingles in the elderly. The vaccine, Zostavax was approved for use in people aged 60 years of age and older (26).

## Rotavirus

Rotavirus is the leading cause of severe diarrhea and vomiting (severe acute gastroenteritis) among young infants and children worldwide. No significant difference was found in the incidence of rotavirus in industrialized and developing countries, suggesting that vaccination may be the only way to control the impact of this severe disease. Dr. Ruth Bishop and colleagues were the first to describe rotavirus in humans in 1973. It was clear, early on, that a naturally acquired first infection, whether symptomatic or asymptomatic, was the most effective defense against severe reinfection, and subsequent infections progressively created greater protection. Therefore, the goal was to create a vaccination that mimicked the effectiveness of naturally acquired immunity following infection. The development of live, attenuated, oral, safe, and effective rotavirus vaccines was then attempted starting in the mid-1970s. Dr. Albert Kapikian and colleagues, at the NIH, developed the RRV strain that was subsequently used to develop the RRV-TV, or the RotaShield, live oral, and tetravalent vaccine licensed in 1998 to be used in infants at 2, 4, and 6 months of age (26). However, due to several reported cases of vaccine-associated intestinal intussusception, RotaShield was pulled off the market in the U.S. 14 months after its introduction on the 16th of October, 1999. In 2004, the National Institute of Allergy and Infectious Diseases (NIAID), part of the NIH, awarded a new license agreement for RotaShield to BIOVIRx, Inc. of Minneapolis, MN, USA, which planned its global commercialization. In 2011, history of intussusception was added as a contraindication for rotavirus vaccination (26). Clark, Offit, and Plotkin then produced the RotaTeq vaccine by Merck based on their bovine strain WC3 in 1992, which was licensed in 2006 by the U.S. FDA. This vaccine, live oral and pentavalent, is destined for use in infants ages 6–32 weeks (26). Another vaccine, Rotarix, was also licensed in 2008. It is a liquid given in a two-dose series to infants from 6 to 24 weeks of age. Before being licensed, both vaccines were shown to be safe and effective in rigorous clinical trials (52).

## Response to Emerging Diseases in the Twenty-First Century

During the past two decades, improvements in environmental health have contributed tremendously to disease vector control. However, substantial challenges remain in dealing with the newly emerging diseases such as severe acute respiratory syndrome (SARS), H1N1, H7N9, and H5N1 influenza, middle east respiratory syndrome (MERS-CoV), rotavirus, Ebola virus, and a variety of other viral, bacterial, and protozoal diseases (53).

The role of vaccines in the control and protection from the above mentioned emerging diseases cannot be overemphasized. Actually, the importance of inducing protective immunity through vaccination came out to be the most powerful tool and effective strategy to prevent the spread of emerging viruses among populations, in particular, among people that are immunologically naïve and susceptible hosts. Such emerging diseases represent a major public health concern; they affect livestock and humans thus threatening the world’s economy and public health. Vaccine strategies for emerging pathologies are limited by sudden appearance of the pathogen and the delayed time consuming traditional vaccine development process. Novel methods to rapidly develop vaccine are being experimented, whereby investigators are working to achieve a better understanding of the nature of the interactions between the immune system and a panel of novel harmful microbes. On this basis several novel strategies have been developed and applied. Such strategies included the use of (1) recombinant proteins, or nanoparticles like in SARS-CoV and MERS-CoV, (2) synthetic peptides like the case of influenza virus, (3) virus-like particles, (4) multimeric presentation of viral antigens like the case of Hepatitis, (5) replication of competent viral vectors like in the Rift Valley Fever virus or the EBOLA viruses, (6) recombinant bacteria for *Listeria* and *Salmonella* among others, and (7) nucleic acid vaccines for EBOLA or Dengue or Rift Valley Fever viruses (54).

In managing or even in preventing the emergence of new infectious diseases, a plan should be developed to strengthen surveillance and promote a multi-partners response within local, national, and global programs.

With the high burden of emerging infectious diseases (EID) it becomes an essential part to find an effective method of either preventing or controlling their spread, that is where the role of vaccines prevails. It is significant to mention that the average case fatality rate for Ebola is around 50% and outbreaks are affecting both developed and developing countries. Another emerging disease, MERS-CoV, has caused the death of around 36% of people reported to have contracted the disease. Another disease with high health and economic burden would be rotavirus which was estimated to have annual direct and indirect costs of around $1 billion with “more than 400,000 physician visits, more than 200,000 emergency department (ED) visits, 55,000 to 70,000 hospitalizations, and 20 to 60 deaths each year in children younger than 5 years” (CDC, 2015). These are few of the facts regarding the affliction of EID most of which have no approved vaccine yet. On the other hand, the influenza virus which was estimated to cause an average of 23,607 deaths annually with a $12 billion cost of an epidemic, showed that with the introduction of its vaccine, studies proved it to be 80% effective in preventing death (55). These figures have managed to influence many governmental and non-profit organizations to intervene either through governmental funding of vaccines where the congress provides yearly international EID funding to several U.S. governmental agencies or through international non-profit organizations which are the leaders in global health innovation (56).

## Disease Burden: Different Influential Aspects

Vaccines remain among the most reliable and effective medical interventions in providing the means to fight debilitating and preventable diseases thus ensuring the continuity of mankind and saving lives. Through reviewing the factsheets provided by the World Health Organization, which provide statistical data on the mortality and morbidity percentages before and after the introduction of the vaccines, one can comprehend the vital role vaccines have played up till this day. Some of the figures that depict the impact of vaccines in decreasing mortality and morbidity include more than 99% decrease in Polio cases since 1988, with cases reaching 350,000 from over 125 endemic countries down to 359 cases as reported in 2014 with only 2 endemic countries, measles vaccine has prevented the death of around 15.6 million children during 2000–2013, in general vaccines prevent around 6 million deaths annually worldwide (57). This success in providing better public health does not negate the economic burden of vaccination. Vaccination programs require excessive funding to ensure proper handling and maintenance of vaccines, adequate staffing and ongoing provision over efficacy and safety of vaccines and the development of newer vaccines (58). Nevertheless, the economic and social burden related to the expenses in hospitalizing affected unvaccinated people still outweighs the aforementioned burden. Moreover, better health in the society would promote economic growth and productivity. Consequently, public awareness and public efforts agree on the importance of vaccination and the implementation of policies regarding mandatory vaccinations as a way to decrease outbreaks of preventable diseases and improve global health and prosperity.

## Turning Point: Vaccine Controversies

As early as the introduction of vaccines, campaigns against vaccination were raging. As with any new medical intervention there are safety concerns that arise which might be deleterious to the public health. Concerns regarding vaccines often follow a path that starts with the hypothesis of a potential adverse event that is impulsively announced to the public without having reproducible studies to confirm this hypothesis, and thus it would take the public several years to regain trust in the vaccine. A notable example in the recent history would be of the paper published by Andrew Wakefield in the British medical journal the *Lancet* in 1998, linking the MMR vaccine to autism. However, his research was discredited and the paper was retracted from the journal after it was proven that actually there is no link between MMR vaccine and autism as per the systemic review by the Cochrane library (59). The battle against vaccines did not reach a halt, and there are still ongoing campaigns that come from religious, political, community-based, and even individual-based grounds raising even ethical issues regarding the mandatory vaccinations proposed by the government. According to the CDC, this year 95% of the children were vaccinated in the U.S., leaving 5% unvaccinated due to religious and philosophical exemptions or even parental refusal due to the fear of vaccine’s side effects and concerns regarding autism from vaccines (60); this is still a critical number since the unvaccinated would pose risk of outbreaks even among the immunized, which necessitates the need for additional awareness campaigns regarding the importance of vaccination since vaccines remain the only plausible measure of protection against preventable diseases. Actually, a trend was reported in the health news lately, in the U.S., that pediatricians refuse to offer medical care for children whose parents declined their vaccination.

## Conclusion

Vaccination has been of great importance throughout centuries (Tables 1 and 2). People started with inoculation techniques dating back to 1000 A.D. with the Chinese, Turks, and Asians. With every century and with every curious physician, inoculation techniques improved gradually giving rise to newer vaccination techniques with Edward Jenner and later on, Louis Pasteur and others. However, there is still plenty of room for improvement with the presence of ongoing epidemics and the spread of newly emerging diseases. One important goal is to strengthen the science base for vaccine development and for public health action and disease prevention. Despite the common belief that infectious diseases were virtually eliminated by the middle of the twentieth century, new and reemerging infections are appearing along with drug resistant infections in the past two decades in the various parts of the world and whose incidence threatens to increase in the near future, due to changes in human demographics and behavior, immigration, and speed of international travel among other things (61–63). The importance of vaccine safety continued to grow throughout the twenty-first century, with the development and licensure of new vaccines added to the already robust immunization armamentarium. Scientists also perfected new ways of administering immunizations including edible vaccines and needleless injections. However, formulated or delivered, vaccines will remain the most effective tool we possess for preventing disease and improving public health in the future. Despite the anti-vaccination campaign and the association of vaccines with some side effects, vaccines continue to remain a cornerstone in global health.

**Table 1:** **Development, introduction, infectious agent, schedule, and efficacy of vaccines**.

Vaccine (year introduced)	Infectious agent	Kind of vaccine first introduced	Vaccine used in present	Time for vaccination	Need of booster	Efficacy
Smallpox (1798)	Variola virus	Live vaccinia virus	N/A	Stopped in 1972 after eradication	N/A	Global eradication
Anthrax (1881)	*Bacillus anthracis*	Live, attenuated	Cell-free filtrates of microaerophilic cultures of a toxigenic, non-encapsulated strainof *B. anthracis* V770-NP1-R	Pre-exposure in adults ≥18 years old; 5 shots over 18 months	Yes; annually	92.50%
Rabies (1884)	Rabies virus	Live, attenuated	Inactivated virus	Post-exposure; 4 doses (0, 3, 7, 14)	Not recommended	Inconclusive data
Typhoid (1896)	*Salmonella typhi*	Inactivated	Inactivated; live, attenuated	At risk population; inactivated: one dose; live, attenuated: 4 doses every other day	Yes if at risk; inactivated: every 2 years; live, attenuated: every 5 years	Varies with age; >50%
Cholera (1884–1896)	*Vibrio cholerae*	Live, attenuated	Oral, inactivated, killed whole cell of *V. cholerae*	At risk population; 2 doses 1 week apart	Yes if at risk; every 6 months	50–60%
Tuberculosis (1927)	*Mycobacterium tuberculosis*	Live, attenuated *Mycobacterium bovis*	N/A	Single dose for children	Not recommended	70–80%
Yellow fever (1935)	Yellow fever virus	Live, attenuated	Live, attenuated	Single dose ≥9 months old	Not recommended	Long term (80–100%)
Diphtheria and tetanus toxoids (1930s and 1940s) and acellular pertussis (dtap)^a^ (1948)	*Corynebacterium diphtheria*, *Bordetella pertussis*, *Clostridium tetani*	Inactivated	Inactivated	2, 4, 6, 15–18 months, 4–6 years	Yes; Tdap: 11–12 years; If Tdap not received between 11–18 years, Tdap dose should be given then followed with Td booster doses every 10 years	80–85%
Poliovirus (1955)	Poliovirus	Inactivated poliovirus	Inactivated and oral, live attenuated	2, 4, 6–18 months	Yes; 4–6 years	IPV: ≥99% OPV: >95%
Influenza (1954–1955)	Influenza virus	Inactivated	Inactivated and live attenuated	Annually	Not recommended	Inactivated: 60% Live attenuated: >87%
Measles, Mumps, Rubella (1971)	Measles, mumps, rubella	Inactivated	Live, attenuated	12–15 months, 4–6 years	Not recommended	>95%
Meningococcal (1974)	*Neisseria meningitidis*	Polysaccharide	Conjugate	11–12 years	Yes; 16 years	>85%
Pneumococcal (1977)	*Streptococcus pneumoniae*	Polysaccharide	Polysaccharide-protein conjugate	2, 4, 6, 12–15 months	Yes; if at risk, ≥65 years old	Children: >90%; adults >65: 75%
Hepatitis B (1981)	Hepatitis B virus	Plasma derived	DNA recombinant	0, 1–2, 6–18 months	Not recommended	Long term (80–100%)
*Haemophilus influenzae* type b (Hib) (1985)	*Haemophilus influenzae* type B	Polysaccharide	Polysaccharide-protein conjugate	2, 4 months	Yes; 12–15months	95–100%
Japanese Encephalitis (1992)	Japanese encephalitis virus	N/A	Inactivated virus	Endemic countries: two-dose series 28 days apart	Yes; if risk of exposure	No data available
Varicella (1995)	Varicella zoster virus	Live	Live, attenuated	12–15 months, 4–6 years	Not recommended	>70%
Hepatitis A (1995)	Hepatitis A virus	Inactivated	Inactivated, whole virus	Two-dose series 6 months apart: 12–23 months old	Not recommended	94–100%
Rotavirus (1998)	Rotavirus	Rhesus-based tetravalent rotavirus	RV1: live, oral, attenuated, monovalent human RV5: live, oral, attenuated, pentavalent bovine-human reassortant	RV1: 2, 4 months RV5: 2, 4, 6 months	Not recommended	85–98%
Human papillomavirus (2006)	Human papillomavirus	DNA recombinant	DNA recombinant	Three-dose series starting at 11 years old: 0, 1–2, 6 months	Not recommended	>99%
Shingles (2006)	Varicella zoster virus	N/A	Live, attenuated	Single dose for people ≥60 years old	Not recommended	97.20%
H1N1 (2009)	Influenza virus type A	N/A	Inactivated virus	Two doses 4 weeks apart for children aged 6 months-9 years or one dose for adults and children >10 years old	N/A	>80%

^a^Upper-case letters denote full-strength doses of diphtheria (D) and tetanus (T) toxoids and pertussis (P) vaccine. Lower-case “d” and “p” denote reduced doses of diphtheria and pertussis used in the adolescent/adult-formulations. The “a” in DTaP and Tdap stands for “acellular.”

*Sources: Ref. (61, 62)*.

**Table 2:** **Development, introduction, infectious agent, schedule, and efficacy of potential new vaccines**.

Potential new vaccines (phase)	Infectious agent	Vaccine used	Time for vaccination	Need of booster	Efficacy
RTS, S/AS01 malaria vaccine (Phase III)	*Plasmodium falciparum*	Malarial circumsporozoite (CS) protein	Children (5–17 months) and young infants (6–12 weeks); 3 doses (months 0, 1, 2)	Yes; month 20	36.30%
Human hookworm vaccine (Phase I)	Nematode parasites *Necator americanus* and *Ancylostoma duodenale*	Recombinant Na-GST-1 (N. Americanusglutathione S-transferase-1) protein	18–45 years old; 3 doses at 56 days intervals	N/A	N/A
Ebola and Marburg vaccine (Phase I)	Ebola and Marburg viruses	Ebola DNA plasmid and Marburg DNA plasmid	18–50 years old; on weeks 0, 4, and 8	N/A	N/A
GBS Vaccine (Phase II)	Group B *Streptococcus*	Polysaccharide capsules from serotypes Ia, Ib, and III of the Group B *Streptococcus*	Females 18–40 years old	N/A	N/A
HIV vaccine (Phase I)	Human immunodeficiency virus	Recombinant gp120	18–50 years old; at months 0, 1, 3, and 6	N/A	N/A
Leishmania Vaccine (Phase III)	Leishmaniasis parasite	Autoclaved *Leishmania* proteins	16–60 years old; 2 doses 30 days apart	N/A	N/A
MERS-cov Vaccine (Phase II)	Middle East respiratory syndrome coronavirus	Purified coronavirus spike protein nanoparticles	N/A	N/A	N/A

*Souce: Ref. (63)*.

The distinctions between national and international health problems are losing ground and could be misleading, the “world is a village.” Clinicians and public health workers need to interact on regular basis with veterinarians and veterinary public health. Actually, good examples of the necessity of such collaboration is the emergence of SARS-CoV and MERS-CoV, it shows clearly how coronaviruses can spillover from animals into humans at any time, causing lethal diseases. Foodborne diseases could lead to regional and international outbreaks which might constitute a threat to national and global security.

## Author Contributions

IH is the first author. She provided the idea and followed along with AJ the execution of the work and final editing. NC and SC did the literature search for the eighteenth and nineteenth century and the respective preliminary writing about this period. SS and RB did the literature search for the twenty-first century, and about general aspects of vaccination and the respective preliminary writing about this period. MR did the literature search regarding vaccine efficacy and effectiveness in the context of the transnational research map and regarding vaccination instruments and inoculating techniques. AG did the literature search and the preliminary writing for the early history of vaccination and wrote a draft of a manuscript about this period. AL edited thoroughly and commented on the final manuscript. AJ supervised the whole process from inception to the final submission and edited the whole manuscript.

## Conflict of Interest Statement

The authors declare that the research was conducted in the absence of any commercial or financial relationships that could be construed as a potential conflict of interest.

## References

[1] Centers for Disease Control and Prevention. Vaccines and Immunizations: The Basics. Washington, DC: Government Printing Office (2012).

[2] CDC.gov Pinkbook. Epidemiology of VPDs, Vaccine Safety. (2015). Available from: http://www.cdc.gov/vaccines/pubs/pinkbook/safety.html

[3] Ellenberg S Chen RT. The complicated task of monitoring vaccine safety. Public Health Rep (1997) 112(1):10–20. 9018282 PMC1381831

[4] Chen RT Hibbs B Vaccine safety: current and future challenges. Pediatr Ann (1998) 27(7):445–55. 10.3928/0090-4481-19980701-11 9677616

[5] NIAID. Topics: Vaccines (2012). Available from: http://www.niaid.nih.gov/topics/vaccines/documents/undvacc.pdf

[6] The College of Physicians of Philadelphia. Articles – Different Types of Vaccines (2014). Available from: http://www.historyofvaccines.org/content/articles/different-types-vaccines

[7] Siegrist CA General aspects of vaccination. 6th ed In: Plotkin SA Orenstein WA Offit PA, editors. Vaccines. Philadelphia, PA: Elseiver Inc (2008). p. 17–36.

[8] Hanekom WA. The immune response to BCG vaccination of newborns. Ann N Y Acad Sci (2005) 1062:69–78. 10.1196/annals.1358.010 16461790

[9] Casadevall A. The methodology for determining the efficacy of antibody-mediated immunity. J Immunol Methods (2004) 291:1–10. 10.1016/j.jim.2004.04.027 15345300

[10] Rimaniol AC Gras G Verdier F Capel F Grigoriev VB Porcheray F Aluminum hydroxide adjuvant induces macrophage differentiation towards a specialized antigen presenting cell type. Vaccine (2004) 22:3127–35. 10.1016/j.vaccine.2004.01.061 15297065

[11] Tan PL Jacobson RM Poland GA Jacobsen SJ Pankratz VS Twin studies of immunogenicity–determining the genetic contribution to vaccine failure. Vaccine (2001) 19:2434–9. 10.1016/S0264-410X(00)00468-0 11257374

[12] Weinberger B Herndler-Brandstetter D Schwanninger A Weiskopf D Grubeck-Loebenstein B. Biology of immune responses to vaccines in elderly persons. Clin Infect Dis (2008) 46(7):1078–84. 10.1086/529197 18444828

[13] Konradsen HB Henrichsen J Wachmann H Holm N. The influence of genetic factors on the immune response as judged by pneumococcal vaccination of mono- and dizygotic Caucasian twins. Clin Exp Immunol (1993) 92:532–6. 10.1111/j.1365-2249.1993.tb03433.x 8513586 PMC1554781

[14] Newport MJ Goetghebuer T Weiss HA Whittle H Siegrist CA Marchant A Genetic regulation of immune responses to vaccines in early life. Genes Immun (2004) 5:122–9. 10.1038/sj.gene.6364051 14737096

[15] Weinberg GA Szilagyi PG Vaccine epidemiology: efficacy, effectiveness, and the translational research roadmap. J Infect Dis (2010) 201(11):1607–10. 10.1086/652404 20402594

[16] Gomez CR Boehmer ED Kovacs EJ. The aging innate immune system. Curr Opin Immunol (2005) 17:457–62. 10.1016/j.coi.2005.07.013 16084711

[17] Curns AT Steiner CA Barrett M Hunter K Wilson E Parashar UD. Reduction in acute gastroenteritis hospitalizations among us children after introduction of rotavirus vaccine: analysis of hospital discharge data from 18 US states. J Infect Dis (2010) 201(10):1617–24. 10.1086/652403 20402596

[18] Clemens JD Shapiro E. Resolving the pneumococcal vaccine controversy: are there alternatives to randomized clinical trials? Rev Infect Dis (1984) 6:589–600. 10.1093/clinids/6.5.589 6390636

[19] Clemens J Brenner R Rao M Tafari N Lowe C. Evaluating new vaccines for developing countries. Efficacy or effectiveness? JAMA (1996) 275:390–7. 10.1001/jama.275.5.390 8569019

[20] Orenstein WA Bernier RH Hinman AR Assessing vaccine efficacy in the field. Further observations. Epidemiol Rev (1988) 10:212–41. 3066628 10.1093/oxfordjournals.epirev.a036023

[21] Szilagyi PG Translational research and pediatrics. Acad Pediatr (2009) 9:71–80. 10.1016/j.acap.2008.11.002 19329097

[22] Kirkup J The Evolution of Surgical Instruments. Novato, CA: Historyofscience.com (2006).

[23] Stanford S. The history of pediatric infectious diseases. Pediatr Res (2004) 55:163–76. 10.1203/01.PDR.0000101756.93542.09 14605240 PMC7086672

[24] The College of Physicians of Philadelphia. History of Vaccines Timelines. The College of Physicians of Philadelphia (1885). Available from: http://www.historyofvaccines.org/content/timelines/all

[25] Marble A Surgeons, Smallpox, and the Poor: A History of Medicine and Social Conditions in Nova Scotia, 1749-1799. Montreal, QC: McGill-Queen’s University Press (1993).

[26] The Immunization Action Coalition. Vaccine Timeline. The Immunization Action Coalition (2013). Available from: http://www.immunize.org/timeline/

[27] Riedel S Edward Jenner and the history of smallpox and vaccination. Proc (Bayl Univ Med Cent) (2005) 18(1):21–5. 16200144 10.1080/08998280.2005.11928028 PMC1200696

[28] Henry EH Experience in Massachusetts and a few other places with smallpox and vaccination. Boston Med Surg J (1921) 185:221–8. 10.1056/NEJM192108251850802

[29] Pepper W The Medical Side of Benjamin Franklin. Philadelphia, PA: W.J. Campbell (1911).

[30] Fenner F History of Vaccine Development (Smallpox Eradication: The Vindication of Jenner’s Prophesy Chapter). New York, NY: Springer Science & Business Media (2011). p. 27–32.

[31] Bazin H History of Vaccine Development. (Pasteur and the Birth of Vaccines Made in the Laboratory Chapter). New York, NY: Springer Science & Business Media (2011). p. 33–45.

[32] Sternbach G. The history of anthrax. J Emerg Med (2003) 24(4):463–7. 10.1016/S0736-4679(03)00079-9 12745053

[33] Bourhy H Perrot A Cavaillon J Vaccines: A Biography (Rabies). New York, NY: Springer Science & Business Media (2010). p. 73–85.

[34] Carpenter C Hornick R Vaccines: A Biography (Killed Vaccines: Cholera, Typhoid, and Plague). New York, NY: Springer Science & Business Media (2010). p. 87–103.

[35] Grabenstein JD Vaccines: A Biography (Toxoid Vaccines). New York, NY: Springer Science & Business Media (2010). p. 105–24.

[36] Cherry JD. Historical review of pertussis and the classical vaccine. J Infect Dis (1996) 174:59–63. 10.1093/infdis/174.Supplement_3.S259 8896526

[37] CDC.gov Preventing tetanus, diphtheria, and pertussis among adolescents: use of tetanus toxoid, reduced diphtheria toxoid and acellular pertussis vaccines recommendations of the advisory committee on immunization practices (ACIP). (2015). Available from: http://www.cdc.gov/mmwr/preview/mmwrhtml/rr5503a1.htm 16557217

[38] Lombard M Pastoret PP Moulin AM. A brief history of vaccines and vaccination. Rev Sci Tech (2007) 26(1):29–48. 17633292 10.20506/rst.26.1.1724

[39] Gheorghiu M Lagranderie M Balazuc AM Vaccines: A Biography (Tuberculosis and BCG Chapter). New York, NY: Springer Science & Business Media (2010). p. 125–40.

[40] Daniel TM The history of tuberculosis. Respir Med (2006) 100(11):1862–70. 10.1016/j.rmed.2006.08.006 16949809

[41] Barrett ADT Higgs S. Yellow fever: a disease that has yet to be conquered. Annu Rev Entomol (2007) 52:209–29. 10.1146/annurev.ento.52.110405.091454 16913829

[42] Monath TP Vaccines: A Biography (Yellow Fever Chapter). New York, NY: Springer Science & Business Media (2010). p. 159–89.

[43] Potter CW A history of influenza. J Appl Microbiol (2001) 91:572–9. 10.1046/j.1365-2672.2001.01492.x 11576290

[44] Artenstein AW Vaccines: A Biography (Influenza Chapter). New York, NY: Springer Science & Business Media (2010). p. 191–205.

[45] Oshinsky D Vaccines: A Biography (Polio Chapter). New York, NY: Springer Science & Business Media (2010). p. 207–21.

[46] Blume S Geesink I A brief history of polio vaccines. Science (2000) 288(5471):1593–4. 10.1126/science.288.5471.1593 10858138

[47] Gallagher KM Plotkin SA Katz SL Orenstein WA Vaccines: A Biography (Measles, Mumps, and Rubella Chapter). New York, NY: Springer Science & Business Media (2010). p. 223–47.

[48] Stern AM Markel H. The history of vaccines and immunization: familiar patterns, new challenges. Health Aff (2005) 24(3):611–21. 10.1377/hlthaff.24.3.611 15886151

[49] Hilleman MR. Vaccines in historic evolution and perspective: a narrative of vaccine discoveries. Vaccine (2000) 18:1436–47. 10.1016/S0264-410X(99)00434-X 10618541

[50] Hilleman MR History of Vaccine Development (Three Decades of Hepatitis Vaccinology in Historic Perspective. A Paradigm of Successful Pursuits Chapter). New York, NY: Springer Science & Business Media (2011). p. 233–46.

[51] Gershon AA Vaccines: A Biography (Varicella and Zoster Chapter). New York, NY: Springer Science & Business Media (2010). p. 265–77.

[52] Dennehy PH Vaccines: A Biography (Rotavirus Chapter). New York, NY: Springer Science & Business Media (2010). p. 347–60.

[53] Fineberg HV Pandemic preparedness and response – lessons from the H1N1 influenza of 2009. N Engl J Med (2014) 370:1335–42. 10.1056/NEJMra1208802 24693893

[54] Hughes JM Emerging infectious diseases: a CDC perspective. Emerg Infect Dis (2001) 7(3):494–6. 10.3201/eid0707.017702 11485640 PMC2631822

[55] World Health Organization. Fact Sheets. World Health Organization (2015). Available from: http://www.who.int/mediacentre/factsheets/en/

[56] KFF.org The U.S. Government & Global Emerging Infectious Disease Preparedness and Response (2015). Available from: http://kff.org/global-health-policy/fact-sheet/the-u-s-government-global-emerging-infectious-disease-preparedness-and-response/

[57] Andre F Booy R Bock H Clemens J Datta S John T Vaccination greatly reduces disease, disability, death and inequity worldwide. Bull World Health Organ (2008) 86(2):140–6. 10.2471/BLT.07.040089 18297169 PMC2647387

[58] Roush S. Historical comparisons of morbidity and mortality for vaccine-preventable diseases in the United States. JAMA (2007) 298(18):2155. 10.1001/jama.298.18.2155 18000199

[59] Demicheli V Rivetti A Debalini M Di Pietrantonj C Vaccines for measles, mumps and rubella in children. Cochrane Database Syst Rev (2012) 19(4):CD004407 10.1002/14651858.CD004407.pub3 22336803 PMC6458016

[60] CDC.gov Vaccination Coverage Among Children in Kindergarten – United States, 2013–14 School Year (2015). Available from: http://www.cdc.gov/mmwr/preview/mmwrhtml/mm6341a1.htm PMC4584748 25321068

[61] Immunize.org Historic Dates and Events Related to Vaccines and Immunization (2015). Available from: http://www.immunize.org/timeline/

[62] CDC.gov Morbidity and Mortality Weekly Report (MMWR) | MMWR (2015). Available from: http://www.cdc.gov/mmwr/index.html

[63] Clinicaltrials.gov Home – ClinicalTrials.gov (2015). Available from: https://clinicaltrials.gov/ct2/home

